# Single cell imaging with near‐field terahertz scanning microscopy

**DOI:** 10.1111/cpr.12788

**Published:** 2020-03-09

**Authors:** Zaoxia Li, Shihan Yan, Ziyi Zang, Guoshuai Geng, Zhongbo Yang, Jiang Li, Lihua Wang, Chunyan Yao, Hong‐Liang Cui, Chao Chang, Huabin Wang

**Affiliations:** ^1^ Center of Applied Physics & Chongqing Engineering Research Center of High‐Resolution and Three‐Dimensional Dynamic Imaging Technology Chongqing Institute of Green and Intelligent Technology Chinese Academy of Sciences Chongqing China; ^2^ College of Instrumentation & Electrical Engineering Jilin University Changchun China; ^3^ Division of Physical Biology CAS Key Laboratory of Interfacial Physics and Technology Shanghai Institute of Applied Physics Chinese Academy of Sciences Shanghai China; ^4^ Bioimaging Center Shanghai Synchrotron Radiation Facility Zhangjiang Laboratory Shanghai Advanced Research Institute Chinese Academy of Sciences Shanghai China; ^5^ Department of Transfusion Medicine Southwest Hospital Third Military Medical University (Army Medical University) Chongqing China; ^6^ Department of Engineering Physics Tsinghua University Beijing China; ^7^ Advanced Interdisciplinary Technology Research Center National Innovation Institute of Defense Technology Beijing China

**Keywords:** dehydration, near‐field imaging, photoconductive antenna microprobe, scanning microscope, single cell, terahertz

## Abstract

**Objectives:**

Terahertz (THz)‐based imaging techniques hold great potential for biological and biomedical applications, which nevertheless are hampered by the low spatial resolution of conventional THz imaging systems. In this work, we report a high‐performance photoconductive antenna microprobe‐based near‐field THz time‐domain spectroscopy scanning microscope.

**Materials and methods:**

A single watermelon pulp cell was prepared on a clean quartz slide and covered by a thin polyethylene film. The high performance near‐field THz microscope was developed based on a coherent THz time‐domain spectroscopy system coupled with a photoconductive antenna microprobe. The sample was imaged in transmission mode.

**Results:**

We demonstrate the direct imaging of the morphology of single watermelon pulp cells in the natural dehydration process with our near‐field THz microscope.

**Conclusions:**

Given the label‐free and non‐destructive nature of THz detection techniques, our near‐field microscopy‐based single‐cell imaging approach sheds new light on studying biological samples with THz.

## 1 INTRODUCTION

Terahertz (THz) radiation refers to the frequency band ranging from 0.1 to 10 THz in the electromagnetic spectrum, corresponding to wavelengths from 3 mm to 30 μm.^1^, ^2^ Recently, THz imaging of biological samples has attracted fast‐growing interest among the scientists in biology‐related fields worldwide due to its unique characteristics such as being highly sensitive to the structure of biomolecules, non‐destructive to biological samples and without the requirement of staining or labelling the samples.^3^, ^4^ For example, THz imaging can provide much more chemical information of a biological sample but has much less ionization damage to the sample than X‐ray microscopy imaging.^5^ For another example, THz imaging can reveal multiple compositional information of a biological sample but has no need for any externally introduced contrast agents or fluorescence probes that are normally required in magnetic resonance imaging or near‐infrared imaging, respectively.^5^, ^6^, ^7^ Consequently, significant progress has been achieved in the study of biological samples with various THz imaging techniques. For example, tissue slices from the lung and breast of patients, and from mouse brain have been successfully investigated using THz imaging techniques, and the diseased regions were unambiguously distinguished from the normal regions for the examined samples.^8^, ^9^, ^10^ One more example, water content and distribution in plant tissues have been successfully investigated using THz imaging techniques.^11^, ^12^ These pioneering studies have manifested the great potential of the application of THz imaging techniques in biological fields such as biomedical detection and plant physiology.

Like biological tissues, cells are also important biological samples that play crucial roles in the life of living organisms. Knowledge on the functional and metabolic activities of cells, particularly of individual cells, is the key to understanding many fundamental biological mechanisms such as disease prevention, development and therapy.^13^ In the past decade, much effort has been spent on exploring the properties of cells using THz‐based techniques. For example, the dielectric constants of several types of human cancer cells were characterized with THz time‐domain spectroscopy (THz‐TDS), and minute changes in the dielectric properties of endothelial cells in response to vascular endothelial growth factor were also measured using a THz‐TDS technique.^14^, ^15^ However, due to the low spatial resolution of conventional THz techniques, limited to the order of the wavelength (0.3 mm@1 THz) according to the Rayleigh criterion, only averaged spectroscopic information of cell monolayer was obtained in these previous studies. Thence, despite of its significance, THz imaging of individual cells has not been documented to date, and it still is a big challenge to image individual cells using THz‐based techniques.^3^

The recently emerged photoconductive antenna microprobe (PCAM)‐based near‐field THz‐TDS scanning microscopy provides researchers good opportunities to interrogate the properties of meta‐materials and semi‐conductors with a spatial resolution far superior to the optical diffraction limit.^16^, ^17^, ^18^ To achieve high‐quality near‐field detection, the PCAM should be positioned as close as possible to the sample surface and the probe‐sample distance should be precisely controlled during the scanning in order to avoid the crash between the probe and sample. Recently, we have successfully imaged mouse brain tissue slices using a home‐built PCAM‐based near‐field THz‐TDS scanning microscope with a calibrated resolution of a few microns.^19^ Unfortunately, imaging individual cells is highly demanded in the imaging speed, signal dynamic range and stability of a system other than the spatial resolution; hitherto, no work has been reported on imaging individual cells using a THz‐based imaging technique. In order to image individual cells, we newly developed a high‐performance (Sections 1‐3 in Supporting Information) PCAM‐based near‐field THz‐TDS scanning system which allows us to collect a full THz waveform in 0.17 seconds with a signal‐to‐noise ratio of ~50 dB, a signal dynamic range of ~50 dB and a calibrated spatial resolution of ~3 μm that is approximately 1‐2 orders higher than that of conventional THz imaging systems. It is expected that such a system could enable us to image single cells.

Cellular activities and functions are closely related to the hydration state of cells since liquid water is the “matrix of life.”^20^ As a result, the hydration state of cells has been intensively investigated using THz‐TDS techniques by measuring cell monolayer to obtain averaged spectroscopic information.^14^, ^20^, ^21^ To make an in‐depth understanding of the hydration properties of cells, however, it is essential to carry out experiments at the single cell level due to the individualism of cells.^22^, ^23^ Observing the natural drying process of biological specimens has been taken as a model system to explore the hydration properties of biological samples. For instance, the dehydration of tissues from plant, cattle, mutton and pork has been investigated using a far‐field THz imaging technique.^11^, ^12^, ^24^

In the present work, we employed our newly established PCAM‐based near‐field THz‐TDS scanning microscope to image individual watermelon pulp cells. The change of the cellular morphology during the natural drying process was successfully monitored, demonstrating that our system has the potential to be used to investigate single cells.

## 2 MATERIALS AND METHODS

### 2.1 Sample preparation

A piece of fresh watermelon pulp was flushed gently by phosphate buffer solution (pH 7.4, ThermoFisher Scientific) with a pipette. The pulp cells flushed out were collected by a Petri dish (60 mm × 15 mm, Corning Inc). Afterwards, a single cell was sucked out by the pipette and deposited onto a 2 cm × 2 cm clean quartz slide (1 mm in thickness), and excess water was blotted off with a paper tower cautiously. Then, the cell was covered by a small piece of PE film (15 μm in thickness) carefully with tweezers to slow down the water evaporation process. The prepared single watermelon pulp cell sample was then transferred to the near‐field THz microscope for test.

### 2.2 Experimental setup

**FIGURE 1:**
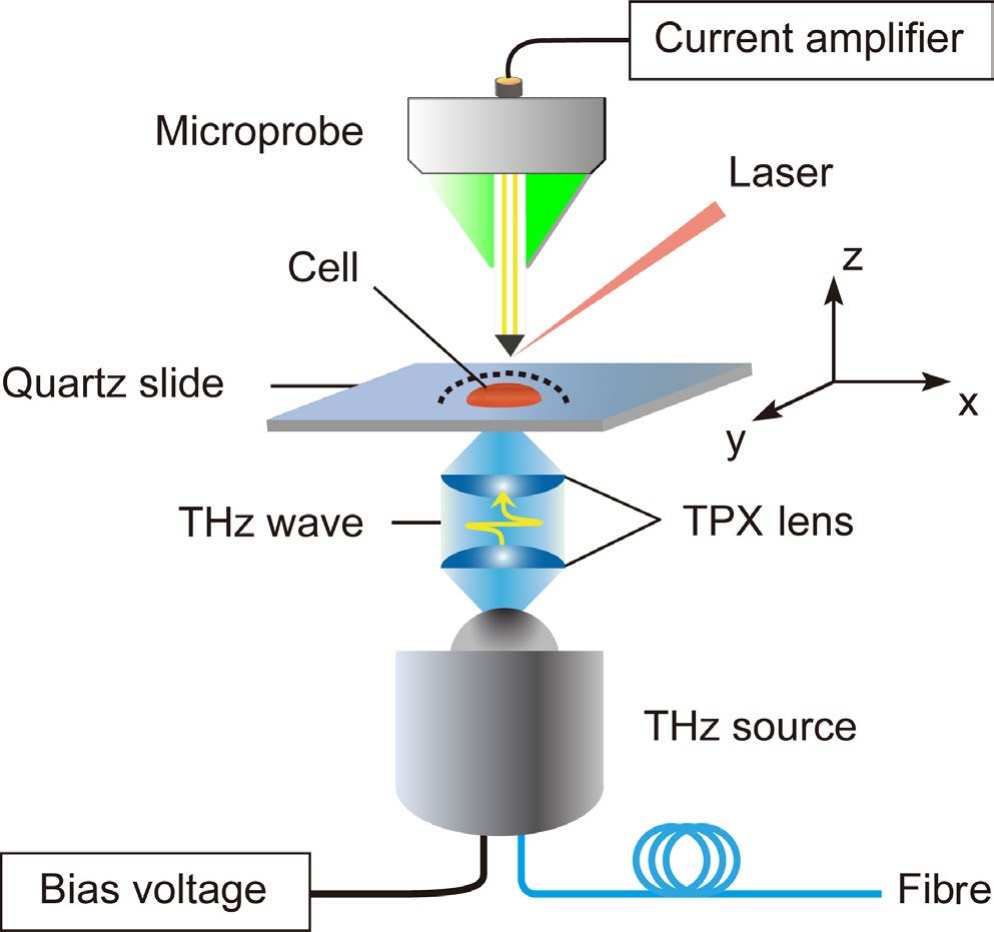
Schematic illustration of the experimental setup. THz radiation emitted from the THz source was firstly collimated by a polymethyl pentene (TPX) lens with an effective focal length of about 50 mm and then focused by another TPX lens onto the back of a quartz slide on which a single watermelon pulp cell (red) was deposited. The diameter of the focused THz radiation spot is about 1 mm@1.0 THz. The near‐field THz signal of the cell was detected by a PCAM detector along the sample surface (indicated by a dashed black line) at a distance of ~10 μm. The sample was controlled to move in the *X*, *Y* and *Z* directions via a precisely controlled 3D translation stage, enabling raster scanning imaging the sample possible

Our near‐field THz‐TDS scanning microscope was based on a coherent THz‐TDS system in transmission mode (Figure 1).^25^, ^26^ The major components of the microscope include a femtosecond laser with a centre wavelength of 780 nm and repetition rate of 100 MHz, a photoconductive antenna THz emitter (THz source), a PCAM (TeraSpike TD‐800‐X‐HR, Protemics GmbH) for near‐field THz detection, collimating and focusing parts for laser and THz optics, a high‐speed optical delay line and a data acquisition system. The laser beam is divided into a pump beam for producing the THz radiation through the antenna emitter and a probe beam for sampling the THz radiation through the PCAM, as what the laser beam is functioning in a conventional THz‐TDS system.^27^ Imaging is realized by raster scanning the sample with the aid of a motorized translation stage (M403.6PD, Physik Instrumente GmbH). The calibrated spatial resolution of the system is ~3 μm, as evaluated by scanning an Au/SiO_2_ grating (Section 2 in Supporting Information). The system allows us to acquire a 90 ps long THz pulse waveform in 0.17 seconds with a signal‐to‐noise ratio and a dynamic range both of ~50 dB. The dynamic range of the signal collected using this system can be better than ~60 dB for an integration time of 3 seconds (Section 3 in Supporting Information). For our previous near‐field system,^19^ it needs ~3 seconds to acquire a 90 ps long THz pulse waveform, and the signal dynamic range can only reach ~45 dB for an integration time of 3 seconds. As a whole, the performance of the new system is much better than our previous near‐field system, particularly in the aspects of imaging speed and signal dynamic range.

### 2.3 THz measurement and data analysis

The prepared sample was loaded on an adjustable aperture iris diaphragm attached to the translation stage. THz radiation was incident from the back of the sample, propagated through the sample and detected by the PCAM positioned above the sample. On the consideration of the dimension of the end of PCAM, the distance between the PCAM and the sample surface was controlled at ~10 μm, in order to avoid any possible crash between the lateral side of the PCAM and the sample. In this situation, the spatial resolution of imaging is ~20 μm when the scanning step size is no larger than 20 μm. The raster scanning step size was set at 20 μm in the *X* and *Y* directions and 1 μm in the *Z* direction. The integration time was set to 1 seconds for each pixel (6 waveforms) for getting enough signal‐to‐noise ratios. It took 1400 seconds (~23 min) to collect an image with a size of 700 μm × 800 μm. The experiments were carried out under a well‐controlled environment with a temperature of 21.0 ± 0.4°C and a humidity of 50 ± 2%. The obtained time‐domain data were transformed into frequency‐domain data by the fast Fourier transform algorithm,^28^ and the electric‐field amplitude was extracted at different frequencies. THz images were constructed from the amplitude data of each pixel at different frequencies. Data analysis was performed using home‐developed codes based on Matlab (Version 2017a, MathWorks Inc).

## 3 RESULTS

### 3.1 High‐performance PCAM‐based THz near‐field microscope

Figure 1 shows a schematic illustration of the experimental setup for the measurement of a single watermelon pulp cell prepared on a hydrophilic quartz slide. Quartz slide is commonly used as the substrate to support the sample being investigated by THz techniques due to its excellent properties in the THz region such as high transmission and low absorption, and it has negligible influence on the experimental results at THz frequencies.^29^ Watermelon pulp cell is very soft and flexible due to its thin (240‐1566 nm), non‐fibrous and highly hydrated cell wall, and can attach to the hydrophilic quartz substrate easily after blotting off the excess water surrounding it.^30^, ^31^, ^32^ A THz radiation beam was focused to the back of the sample, and a PCAM detector was aligned above the sample to detect the near‐field THz signal. With the aid of a 3D translation stage, the sample could be controlled to move in the *X*, *Y* and *Z* directions with a precision of 1 μm. Cell imaging was realized by raster scanning the sample, and the distance between the sample surface and the PCAM was kept at ~10 μm during the scanning. To avoid rapid drying of the cell, the cell was covered with a thin polyethylene film (~15 μm in thickness) that has negligible influence on the THz radiation.^33^

### 3.2 Images of a single watermelon pulp cell at different THz frequencies

**FIGURE 2:**
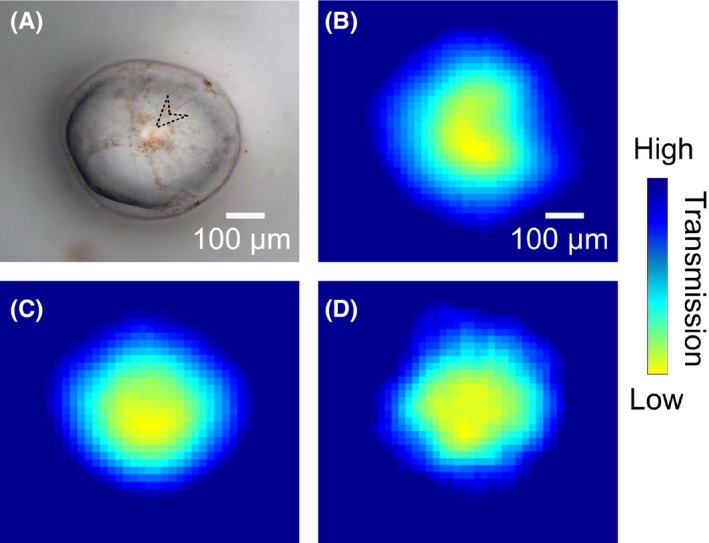
Images of a single watermelon pulp cell. A, Optical image, and B‐D, THz amplitude images at 0.88, 1.66 and 2.13 THz, respectively. The arrow head in (A) indicates the region where the cell nucleus locates

Figure 2 shows the optical image and corresponding THz images at different frequencies of a single watermelon pulp cell. Watermelon pulp cell is a type of parenchyma cell, and the optically evident dense region indicated by an arrow head in Figure 2A can be assigned to the region where the cell nucleus locates.^30^, ^34^, ^35^ It can be found that the THz images of the cell at different frequencies have different qualities. The image at 1.66 THz (Figure 2C) has the best quality compared to that at 0.88 THz (Figure 2B) or 2.13 THz (Figure 2D) because it is the one that is most consistent with the optical image of the cell (Figure 2A) in terms of the cellular size and shape. A lower frequency THz wave (eg 0.88 THz) could lead to a stronger optical diffraction effect due to longer wavelength, which can distort the THz image. However, a higher frequency (eg 2.13 THz) means a lower signal‐to‐noise ratio for a THz‐TDS system, which can result in poor quality of the image due to high noise levels. Therefore, a suitable frequency should be chosen to construct THz images, which is a common practice in THz imaging and dependent on the measured sample as well.^12^, ^36^ In our case, the image constructed from the data at 1.66 THz can give the best result.

All THz images present that THz transmission is reduced gradually from the edge to the central area of the cell, indicating increasing THz absorption from the rim to the middle of the cell. Since the materials contained in the cell absorb THz radiation and the height of the cell is increasing from the border (~0 μm) to the central region (~150 μm) gradually, it is reasonable to observe the above results. According to the contrast of the THz images, the cell can be roughly divided into three regions, that is the region around the cell wall/membrane (blue colour), the region around the cell nucleus (yellow colour) and the region (cyan colour) between the previously mentioned two regions.

### 3.3 THz images of a single watermelon pulp cell during the drying process

**FIGURE 3:**
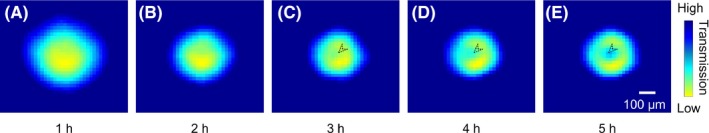
THz images of a single watermelon pulp cell during the drying process. In general, the size of the cell shown in THz images (A‐C) became smaller, and the arrow head in (C‐E) indicates the location where the colour changes from yellow (C) to cyan (D) and to blue (E) with time. The images were constructed from the data at 1.66 THz

It is clearly observed from Figure 3 that the size of THz images of the cell became smaller and smaller for the first three hours (Figure 3A‐C), but changed slightly for the later three hours (Figure 3C‐E). This can be interpreted as that for the first three hours the cell shrank quickly due to fast water‐loss, but three hours later the peripheral shrinking of the cell became much slower than the initial three hours due to decreased water evaporation rate.^12^ The above‐mentioned tendency has also been proved by visible optical imaging. Importantly, it was observed that the THz transmission in the most inner area of the cell increased with time from 3 to 5 hours (Figure 3C‐E), indicated by arrow heads. The above results indicate that during the natural drying process, the structure in the cell was changed.^37^

## 4 DISCUSSION

**FIGURE 4:**
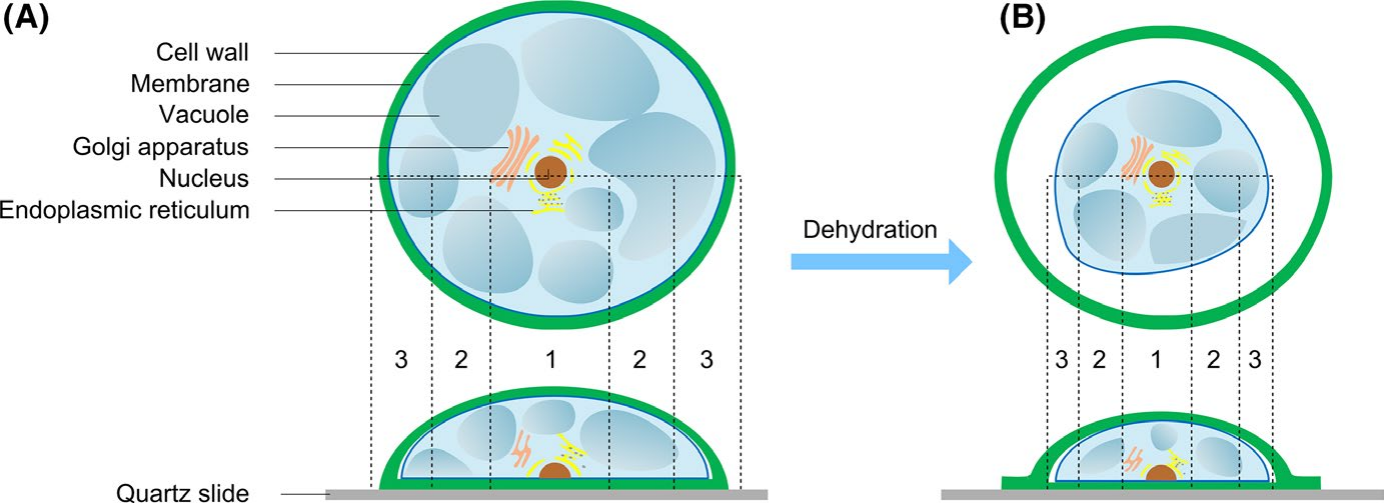
A cartoon showing the structure of a watermelon pulp cell. A, The top and bottom panels show the top view and side view of the cell initially adhered to a quartz slide, respectively. The cell can be approximately divided into three regions: region “1” is around the membrane, region “3” around the nucleus, and region “2” between the former mentioned two regions. Some cellular organelles are presented. B, Top and side view of the cell in partly dehydration. The plasma membrane separates from the cell wall (top view), and at the cellular periphery, the cell wall overlaps with each other due to the lack of the support from cytoplasm (side view). The overlapped cell wall is very thin and cannot be detected in THz imaging

A cartoon showing the structure of the watermelon pulp cell (Figure 4) was sketched to help interpret the phenomena observed during the cell drying process. Briefly, the cell is mainly composed of the cell wall, plasma membrane, nucleus and cytoplasm. Except for the nucleus, the cytoplasm includes all materials within the membrane such as water, vacuoles, Golgi apparatus, and endoplasmic reticulum. Besides the above information, some other essential information for a normal watermelon pulp cell that should be known includes the following: (a) the nucleus occupies a small volume portion of the cell, and vacuoles account for most of the cell volume; and (b) vacuoles are rich in water.^38^, ^39^

When a cell is initially laid on the substrate (a quartz slide in our case), it can be roughly divided into three regions (Figure 4A). The central region has the most height (region 1), the region around the membrane has the least height (region 3), and the region between the region 1 and the region 3 has a medium height (region 2). When dehydration occurs due to water evaporation, changes will happen to the cell (Figure 4B), such as vacuole collapse, separation between plasma membrane and cell wall, and cytoplasm/cell shrinking.^40^

Since we used a transmission mode imaging technique, the size of the THz image of the cell is related to the projected area of the cell/cytoplasm on the substrate while the contrast of the THz image is related to both the components and height of the cell. For a cell in a well hydration state, a THz radiation needs to travel the longest distance in region 1 and the least distance in region 3 before it transmits through the cell. Because water has a very strong THz absorption ability (~220 cm^−1^@1 THz)^41^, ^42^ and is the predominant component in all these regions, so the power‐loss of THz radiation transmitted through the cell is in the sequence: region 1 > region 2 > region 3. This is consistent with the THz images obtained at the 1st hour and the 2nd hour when the cell was in good hydration states. The size of the THz image of the cell at the 2nd hour is smaller than the 1st hour can be ascribed to water‐loss‐induced cellular contraction.

Compared to the 2nd hour, the size of THz image of the cell at the 3rd hour further shrank due to further water evaporation. But from the 3rd hour onwards (the 4th hour and 5th hour), the size change of THz images of the cell was observed slightly, possibly due to slow water‐loss. It needs to note that although the projected areas of the cell were changing slowly during this period of time, as indicated by the cellular size in the THz images, the height of the cell might continue to change obviously due to water evaporation.^43^ Interestingly, it was observed that the colour of the innermost of the corresponding three images changed gradually from yellow to blue, indicating continuous height decrease of the cell. These results implied very meaningful information on the structural change in the cell during the natural drying process. On one hand, after a rapid water‐loss in the first three hours the rate of water‐loss was slowed down and the cellular peripheral contraction was obviously decreased. On the other hand, the influence of the nucleus could not be neglected anymore on the THz absorption from the 3rd to the 5th hours when substantial amount of the water had evaporated to decrease the cell height and the volume (mass) ratio of nucleus (possibly surrounded with some other cellular organelles) to water could no longer be neglected. Since the nucleus (together with the possibly surrounded cellular organelles) is a structure rich of biomolecules and the THz absorption by biomolecules is an order of magnitude lower than water,^44^, ^45^, ^46^ increased volume ratio of nucleus (and other possibly surrounded cellular organelles) to water can lead to a decreased THz absorption, namely, increased THz transmission. During the period of the last three hours (hour 3‐5), the effect of nucleus region on THz absorption was getting more and more notable with continued water‐loss, consequently, resulting in the observed colour change in the THz images.

Finally, it might be necessary to note that although our microscope can be used to scan along the cell topographical surface it can only perform two‐dimensional imaging at this stage. With further development of THz source, detector and imaging theories, our near‐field THz scanning microscope can be possibly updated to a THz microscope that has the ability to obtain reliable three‐dimensional (3D) images of cells and other biological samples, so as to provide more detailed information on the internal structure of the samples.^47^

In summary, we have developed a PCAM‐based near‐field THz‐TDS scanning microscope, by which the morphological change of single watermelon pulp cell was successfully investigated during the cellular drying process. This initial work demonstrates that it is possible to investigate single cell with our THz imaging technique. Since this is the first time to image single cells, we chose an easy handling watermelon pulp cell as the model cell. The present work may be regarded as a good start point for single‐cell THz imaging. With more input from the scientific communities such as further miniaturization of the PCAM, the application of high power broad‐band THz source, and the establishment of THz spectral database of biological compositions, logarithms for biological component analysis and theories for 3D imaging, we believe that the PCAM‐based near‐field THz scanning microscopy has the potential to be exploited to reveal physiological states (eg normal or diseased), subcellular structural change, biological activities and cellular heterogeneities of various types of cells (plant cell, animal cell and bacterial cell).^48^ In addition, this technique can also be employed to detect tissue samples to disclose more detailed information than traditional THz imaging techniques. As a label‐free, non‐destructive and sensitive biological detection technique, the demonstrated THz imaging technique will find numerous important applications in the field of THz biotechnology.

## CONFLICT OF INTEREST

The authors declare no competing financial interest.

## AUTHOR CONTRIBUTION

HW, SY and CY conceived the original idea; ZL, SY and HW designed the experiment; HW and C Y supervised the project; ZL, SY and ZZ performed the experiment; ZL, SY, ZY and HW analysed the data; ZL, GG, HW and H‐LC developed the instrument; and ZL, SY, HW, JL, LW and CC produced the manuscript. All the authors participated in discussions and reviewed the manuscript.

## Supporting information

Supplementary Material

## Data Availability

All data generated or analysed during this study are available in this article.
