# Supplementary material for: Single cell imaging with near‐field terahertz scanning microscopy
Source: Cell Prolif. 2020 Mar 9;53(4):e12788. doi: 10.1111/cpr.12788 (PMC7162806; doi:10.1111/cpr.12788)
Supplement: Supplementary file 1 — Supplementary Material [file CPR-53-e12788-s001.docx]

Supporting Information for “**Single cell imaging with near-field terahertz scanning microscopy**”

Zaoxia Li,^1, 2 #^ | Shihan Yan,^1 #^ | Ziyi Zang,^1, 2^ | Guoshuai Geng,^1, 2^ | Zhongbo Yang,^1^ | Jiang Li,^3, 4^ | Lihua Wang,^3, 4^ | Chunyan Yao,^5*^ | Hong-Liang Cui,^1, 2^ | Chao Chang ^6, 7^ | Huabin Wang ^1*^

^1^Center of Applied Physics & Chongqing Engineering Research Center of High-Resolution and Three-Dimensional Dynamic Imaging Technology, Chongqing Institute of Green and Intelligent Technology, Chinese Academy of Sciences, Chongqing, 400714, China

^2^College of Instrumentation & Electrical Engineering, Jilin University, Changchun, 130061, Jilin, China

^3^Division of Physical Biology, CAS Key Laboratory of Interfacial Physics and Technology, Shanghai Institute of Applied Physics, Chinese Academy of Sciences, Shanghai, 201800, China

^4^Bioimaging Center, Shanghai Synchrotron Radiation Facility, Zhangjiang Laboratory, Shanghai Advanced Research Institute, Chinese Academy of Sciences, Shanghai, 201210, China

^5^Department of Transfusion Medicine, Southwest Hospital, Third Military Medical University (Army Medical University), Chongqing 400038, China

^6^Department of Engineering Physics, Tsinghua University, Beijing, 100084, China

^7^Advanced Interdisciplinary Technology Research Center, National Innovation Institute of Defense Technology, Beijing, 100071, China

^#^ Zaoxia Li and Shihan Yan should be considered joint first author.

***Correspondence**

*Huabin Wang (*[*wanghuabin@cigit.ac.cn*](mailto:wanghuabin@cigit.ac.cn)*) or Chunyan Yao (yaochunyan@tmmu.edu.cn)*

### 1 | Development of High Performance PCAM-based Near-Field THz-TDS Scanning System

In our present work, we developed a high performance PCAM-based THz near-field scanning system by adopting several technical strategies, mainly including: (1) adopting a magnet motor in the new system to replace the voice coil motor in the old system to speed the data acquisition; and (2) using optical fiber-based optical paths and new-branded high quality laser in the new system to replace the free space-based optical paths and old-branded laser in the old system to improve the stability, signal to noise ratio and dynamic range.

### 2 | Verification of the Spatial Resolution of the System

An Au/SiO_2_ grating with a period of 10 μm was used as the standard sample for the test of the spatial resolution of the system. The grating consists of a 1 mm thick SiO_2_ substrate coated with 50 nm thick Au strips. The influence of the gold height on THz signal is negligible for such a thin gold film. Line scan was performed across the grating to obtain the THz signal that was plotted as “transmission amplitude vs. distance”, as shown in Figure S1. During the measurement, the scanning step was set to 1 μm and the PCAM tip-sample separation was controlled to within 3 μm. The experiments were carried out under a well-controlled environment with a temperature of 21.0 ± 0.4°C and a humidity of 50 ± 2%. According literature,^1^ the distance for the transition region between 90% transmission and 10% transmission can be defined as the spatial resolution, which is 3 μm for our system, as marked in Figure S1, verifying that the nominal spatial resolution of the near-field THz microscope is ~ 3 μm.


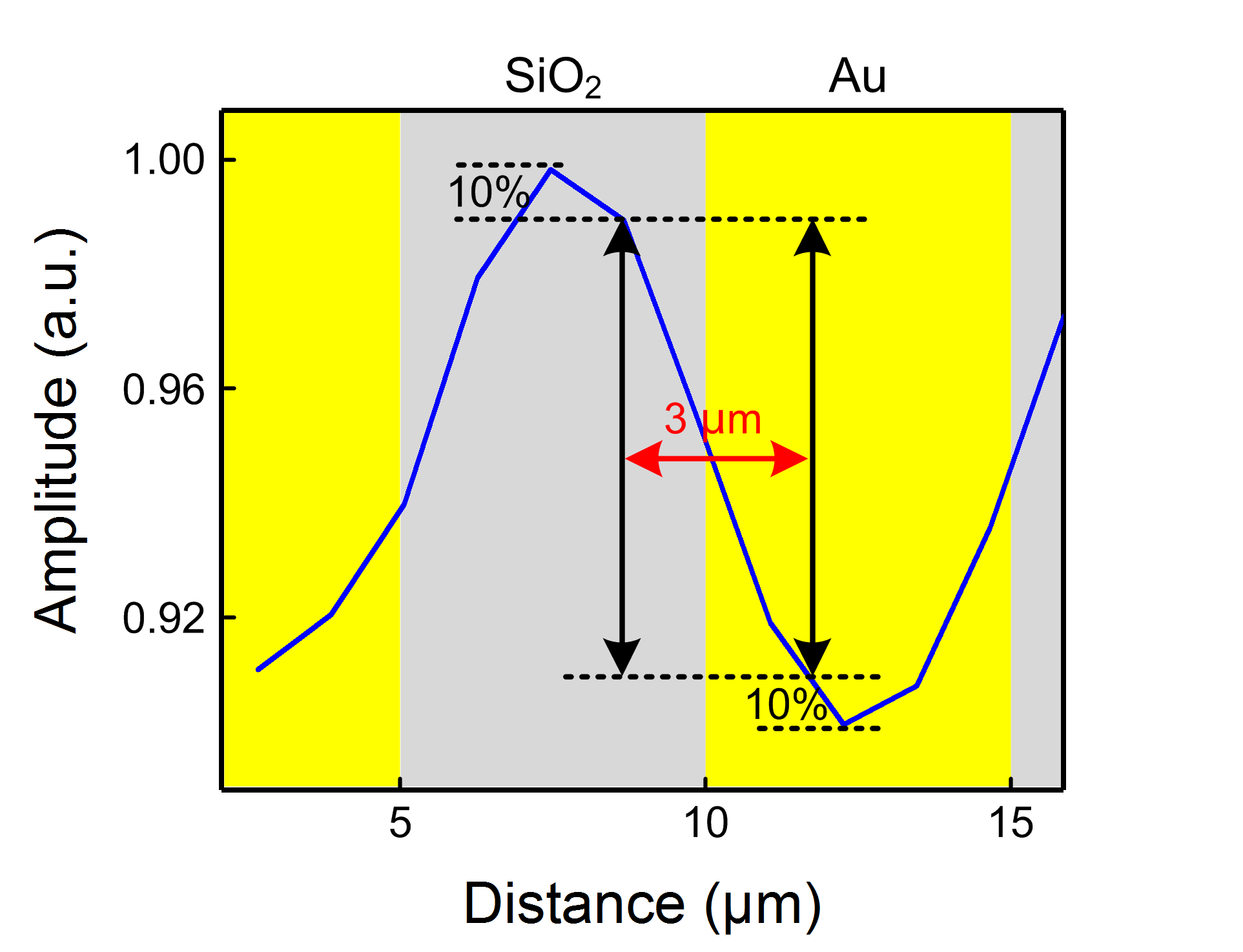


**FIGURE S1** Verification of the spatial resolution of the near-field THz imaging system.

### 3 | Assessment of the Quality of the Spectroscopy

For our near-field THz-TDS scanning imaging system, the quality of the spectroscopy is the basis for achieving high-quality images in terms of accuracy, dynamic range and stability. To evaluate the quality of the spectroscopy, a PCAM was used to collect spectra in air at a temperature of 21.0 ± 0.4°C and a humidity of 50 ± 2%. As an example, the obtained spectroscopy with an integration time of 200 s is shown in Figure S2, from which it can be seen that (1) the dynamic range can reach ~ 85 dB; and (2) the typical absorption peaks of water vapor are highly consistent with the literature.^2^ The results confirm the high spectroscopy quality of our near-field system. We have tested the quality of the spectra for different integration durations, and they are all in good quality. The dynamical ranges of some of the collected spectra are listed in Table S1.


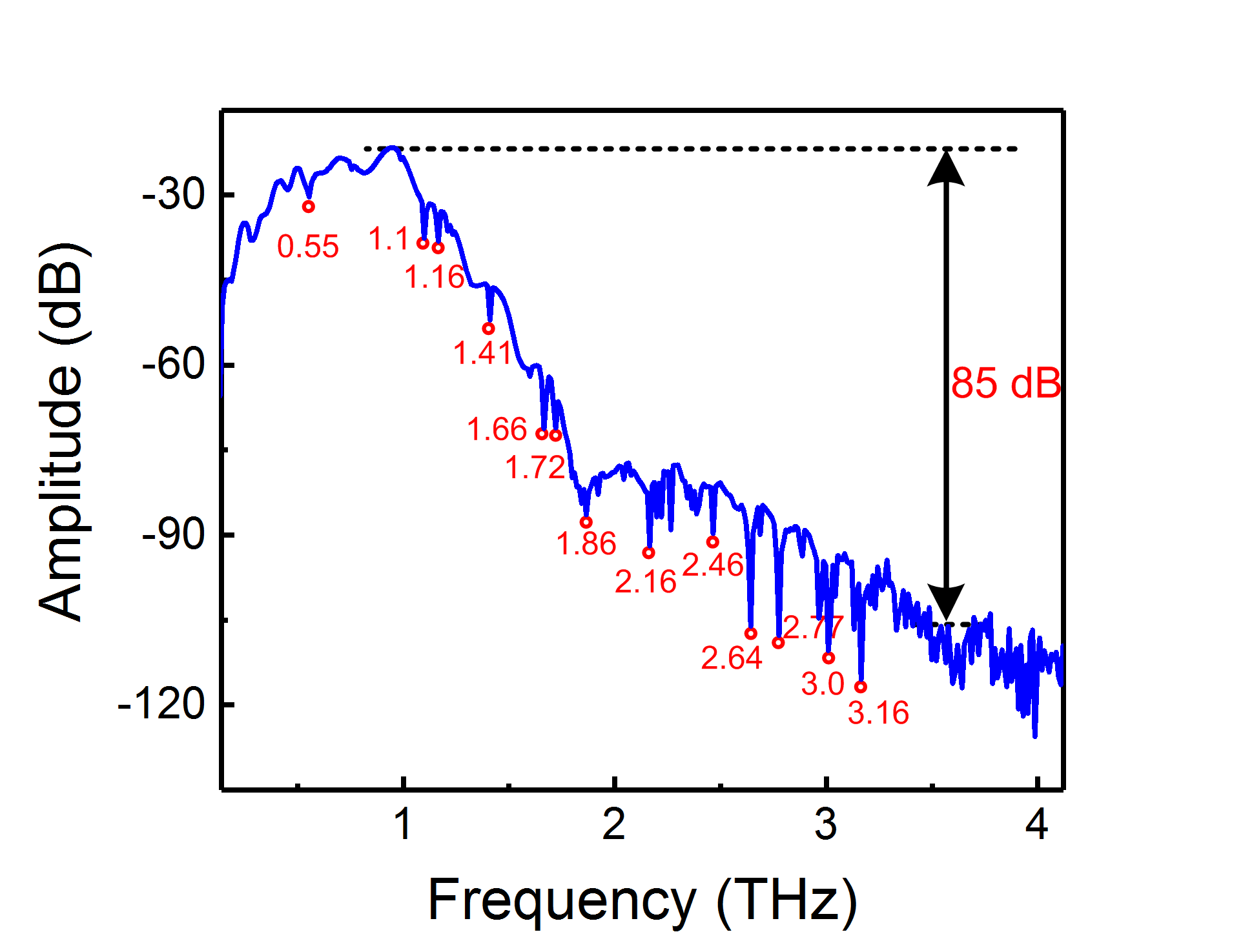


**FIGURE S2** Typical absorption peaks of water vapor between 0.1 - 3.5 THz.

**TABLE S1** The dynamic range of the spectra for different integration durations.

| Integration Time (s) | 1 | 2 | 5 | 10 | 20 | 50 | 100 | 200 |
| --- | --- | --- | --- | --- | --- | --- | --- | --- |
| Dynamic Range (dB) | 54 | 60 | 66 | 68 | 70 | 76 | 80 | 85 |

## REFRENCES

1. Wächter M, Nagel M, Kurz H. Tapered photoconductive terahertz field probe tip with subwavelength spatial resolution. *Appl Phys Lett.* 2009;95(4):041112.
2. Cui H, Zhang XB, Yang YX, et al. Vibration-rotation absorption spectrum of water vapor molecular in frequency selector at 0.5-2.5 THz range. *Optik* 2015;126(23):3533-3537.
